# Supplementary material for: Four Core Genotypes mice harbour a 3.2MB X-Y translocation that perturbs Tlr7 dosage
Source: Nat Commun. 2024 Oct 11;15:8814. doi: 10.1038/s41467-024-52640-8 (PMC11470063; doi:10.1038/s41467-024-52640-8)
Supplement: Supplementary file 1 — Supplementary Information [file 41467_2024_52640_MOESM1_ESM.pdf]

Four-Core Genotypes mice harbour a 3.2MB X-Y translocation that perturbs *Tlr7* dosage.

Supplementary Table 1: Publications using FCG mice. The following studies have made use of the FCG mouse model. We emphasise that the translocation does not necessarily affect the conclusions drawn in each of them, as many of them dissect phenotypes unaffected by the translocation and/or they validate results with orthogonal approaches, including other mouse models. Finally, the colonies used in these studies (e.g., MF1 FCG, see Supplementary Figures 14 and 15) might not necessarily carry the translocation.

**Authors and year**	**Digital object identifier (DOI)**	**Context**
De Vries et al, 2002	10.1523/JNEUROSCI.22-20-09005.2002	Brain
Carruth et al, 2002	10.1038/nn922	Brain
Xu et al, 2002	10.1093/hmg/11.12.1409	Brain
Ishikawa et al, 2003	10.1095/biolreprod.102.012641	Placenta
Markham et al, 2003	10.1016/S0306-4522(02)00554-7	Brain
Durcova-Hills et al, 2004	10.1016/j.ydbio.2003.12.018	Primordial germ cells
Wagner et al, 2004	10.1210/en.2003-1219	Brain
Palaszynski et al, 2005	10.1210/en.2005-0284	Immune system
Xu et al, 2005	10.1111/j.1460-9568.2005.04134.x	Brain
Xu et al, 2005	10.1002/jnr.20429	Brain
Xu et al, 2005	10.1080/07435800500229243	Kidney
Gatewood et al, 2006	10.1523/JNEUROSCI.3743-05.2006	Brain
Xu et al, 2006	10.1016/j.modgep.2005.06.011	Brain
Quinn et al, 2007	10.1038/nn1994	Brain
Chen et al, 2008	10.1002/dneu.20581	Neural Tube
Gioiosa et al, 2008	10.1016/j.jpain.2008.06.001	Nervous system
Gioiosa et al, 2008	10.1016/j.yhbeh.2007.09.003	Nervous system
McPhie-Lalmansingh et al, 2008	10.1016/j.yhbeh.2008.05.016	Brain
Park et al, 2008	10.1111/j.1601-183X.2008.00397.x	Brain
Smith-Bouvier et al, 2008	10.1084/jem.20070850	Immune system
Xu et al, 2008	10.1523/JNEUROSCI.5382-07.2008	Brain

Xu et al, 2008	10.1371/journal.pone.0002553	Brain
A van Nas et al, 2009	10.1210/en.2008-0563	Adipose, liver, skeletal muscle, brain
Chen et al, 2009	10.1111/j.1460-9568.2009.06610.x	Brain
Ji et al, 2010	10.1161/HYPERTENSIONAHA.109.144949	Cardiovascular system
Kuo et al, 2010	10.1186/2042-6410-1-7	Brain
Liu et al, 2010	10.1186/2042-6410-1-6	Kidney
Barker et al, 2010	10.1523/JNEUROSCI.0548-10.2010	Brain
Wijchers et al, 2010	10.1016/j.devcel.2010.08.005	Thymus
Abel et al, 2011	10.1159/000324402	Brain
Caeiro et al, 2011	10.1161/HYPERTENSIONAHA.111.175661	Heart
Cox et al, 2011	10.1111/j.1601-183X.2011.00688.x	Brain
Robinson et al, 2011	10.1186/2042-6410-2-8	Immune system
Bonthuis et al, 2012	10.1016/j.yhbeh.2012.02.003	Brain
Chen et al, 2012	10.1371/journal.pgen.1002709	Fat, Liver
Sasidhar et al, 2012	10.1136/annrheumdis-2011-201246	Spleen, kidney
Kuljis et al, 2013	10.1210/en.2012-1921	Brain
Chen et al, 2013	10.1210/en.2012-2098	Fat
Ehlen et al, 2013	10.1371/journal.pone.0062205	Brain
Kopsida et al, 2013	10.1371/journal.pone.0073699	Brain
Moore et al, 2013	10.1016/j.neuroscience.2013.04.017	Brain
Seney et al, 2013	10.1186/2042-6410-4-20	Brain
Seney et al, 2013	10.3389/fpsyt.2013.00104	Brain
Dadam et al, 2014	10.1152/ajpregu.00447.2013	Brain
Li et al, 2014	10.1093/cvr/cvu064	Heart
Scerbo et al, 2014	10.3389/fncel.2014.00188	Brain
Seu et al, 2014	10.1111/gbb.12143	Brain
Chen et al, 2015	10.1016/j.yhbeh.2015.07.020	Brain, adipose
Cisternas et al, 2015	10.1016/j.mce.2015.07.027	Brain

Link et al, 2015	10.1161/ATVBAHA.115.305460	Blood, liver
Manwani et al, 2015	10.1038/jcbfm.2014.186	Brain
Pessôa et al, 2015	10.1161/HYPERTENSIONAHA.115.05303	Cardiovascular system
Puralewski et al, 2015	10.1186/s13293-016-0106-6	Brain
Quinnies et al, 2015	10.1186/s13293-015-0026-x	Brain
Vivas et al, 2015	10.1016/j.physbeh.2015.08.010	Heart, brain, kidney
Corre et al, 2016	10.1007/s00429-014-0952-0	Brain
McCullough et al, 2016	10.18632/aging.100997	Brain
Cambiasso et al, 2017	10.1080/01677063.2017.1390572	Brain
Cisternas et al, 2017	10.1038/s41598-017-05658-6	Brain
Dadam et al, 2017	10.1016/j.mce.2017.02.041	Brain, kidney
Link et al, 2017	10.1186/s12864-017-3484-1	Adipose
Kaneko et al, 2018	10.1126/sciadv.aar5598	Bladder
Vousden et al, 2018	10.1016/j.neuroimage.2018.02.051	Brain
Barko et, 2019	10.1159/000499105	Brain
Davis et al, 2019	10.1111/acel.12871	Whole animal (ageing)
Dill-Garlow et al, 2019	10.1038/s41598-018-37175-5	Lymph nodes
Golden et al, 2019	10.1073/pnas.1910072116	Immune system
Itoh et al, 2019	10.1172/JCI126250	Immune system
Cisternas et al, 2020	10.1038/s41598-020-65183-x	Brain
Martini et al, 2020	10.1016/j.yhbeh.2020.104821	Brain
Aarde et al, 2021	10.1111/gbb.12685	Brain
Doss et al, 2021	10.1016/j.celrep.2021.108833	Immune system
Ensor et al, 2021	10.1101/2021.09.14.460275	Lung
Gata-Garcia et al, 2021	10.3389/fneur.2021.721108	Brain
Ghosh et al, 2021	10.3389/fendo.2021.582614	Immune system
Grimm et al, 2021	10.1172/jci.insight.146863	Lung
Qi et al, 2021	10.1186/s12974-021-02120-3	Brain
Shi et al, 2021	0.1016/j.devcel.2021.09.022	Heart

Zapata et al, 2021	10.1007/s00018-021-03945-0	Brain
Aarde et al, 2022	10.1101/2022.10.25.513748	Brain
Blencowe et al, 2022	10.1101/gr.275965.121	Liver, adipose
Broestl et al, 2022	10.1038/s42003-022-03743-9	Brain
Ocañas et al, 2022	10.1007/s12035-022-02860-0	Brain
Sneddon et al, 2022	10.1111/adb.13222	Brain
Taylor et al, 2022	10.1002/jnr.24704	Nervous system
Wiese et al, 2022	10.1186/s13293-022-00474-8	Liver, blood
Zhang et al, 2022	10.1101/2022.08.29.505759	Cardiovascular system
Cheng et al, 2023	10.1038/s41590-023-01463-8	Immune system
Lopez-Lee et al, 2023	10.1101/2023.09.19.558439	Brain
Le et al, 2023	10.1159/000531648	Brain
Sneddon et al, 2023	10.3389/fpsyt.2023.1098387	Brain
Dhakal et al, 2023	10.1101/2023.11.27.568847	Immune system
Sakamuri et al, 2023	10.1530/mah-23-0010	Gut
Sakamuri et al, 2023	10.21203/rs.3.rs-3370040/v1	Cardiovascular system
Commodore et al, 2024	10.1152/physiolgenomics.00049.2023	Lung
Ekpruke et al, 2024	10.1152/physiolgenomics.00112.2023	Lung
Cioffi et al, 2024	10.1016/j.jsbmb.2024.106514	Brain
Amato-Menker et al, 2024	10.1186/s13293-024-00597-0	Immune system

Supplementary Table 2: Expression and overexpression phenotype of translocation genes. Gene functions were obtained from UniProt (https://www.uniprot.org/). Cell types with gene upregulation were determined by browsing the Tabula Muris FACS-based scRNA-seq dataset (https://tabula-muris.ds.czbiohub.org/) and listed in decreasing order of expression. Functional consequences of increased gene expression were identified in the cited publications.

**Gene name and function**	**Cell types where the gene is robustly expressed**	**Functional consequences of increased gene expression**
Hccs<br>(lyase that catalyzes the covalent linking of the heme group to the cytochrome C apoprotein to produce the mature functional cytochrome)	Heart cardiac muscle cells, bone marrow late pro-B cells, bone marrow pre-natural killer cells, bladder urothelial cells, bone marrow basophils, brain oligodendrocyte precursor cells, skin epidermal stem cells, bone marrow Slamf1-positive multipotent progenitor cells, brain oligodendrocytes, bone marrow granulocytopoetic cells, bone marrow granulocyte monocyte progenitor cells, bone marrow common lymphoid progenitors, trachea blood cells, bone marrow mature nature killer cells, bone marrow B cells, pancreatic PP cells, bone marrow megakaryocyte-erythroid progenitor cells, skin leukocytes, pancreatic beta cells, tongue epidermal basal cells, tongue keratinocytes, pancreatic alpha cells, limb muscle T cells, brain pericytes, thymus DN1 pro-T cells, large intestine epithelial cells, liver hepatocytes, brain neurons, mammary gland basal cells, bone marrow monocytes, bone marrow Slamf1-negative multipotent progenitor cells, skin epidermal cells, large intestine goblet cells, pancreatic delta cells	
Amelx<br>(protein involved in the biomineralization of teeth)	None	Cho et al, 2014[35]; Miao et al, 2021[36]

Arhgap6 (GTPase activator for the Rho-type GTPases by converting them to an inactive GDP-bound state)	Heart myofibroblast cells, brain pericytes, pancreatic PP cells, bone marrow basophils, bladder cells, pancreatic stellate cells, bone marrow common lymphoid progenitors, pancreatic alpha cells, bone marrow hematopoetic precursor cells, bone marrow megakaryocyte-erythroid progenitor cells, heart smooth muscle cells, lung smooth muscle cells, Slamf1-positive multipotent progenitor cells, mammary gland stromal cells, fat mesenchymal stem cells	Prakash et al, 2000[37]; Wu et al, 2019[38]; Li et al, 2020[39]; Chen et al, 2023[40]
Msl3 (component of the MSL complex that acetylates histone H4, remodels chromatin and regulates transcription)	Bone marrow pre-natural killer cells, bone marrow Slamf1-positive multipotent progenitor cells, bone marrow common lymphoid progenitors, skin epidermal stem cells, bone marrow megakaryocyte-erythroid progenitor cells, bone marrow late pro-B cells, bone marrow granulocyte-monocyte progenitor cells, lung ciliated columnar cells of tracheobronchial tree, cardiac muscle cells, bone marrow Slamf1-negative multipotent progenitor cells, large intestine epithelial cells, bone marrow granulocytopoetic cells, tongue epidermal basal cells, large intestine goblet cells, brain pericytes, skin epidermal cells, liver hepatocytes, bladder urothelial cells, bone marrow basophils, fat mesenchymal stem cells, tongue keratinocytes, pancreatic ductal cells, bone marrow hematopoetic precursor cells, thymus DN1 thymic pro-T cells, skin epidermal basal cells, bone marrow monocytes, bone marrow macrophages, heart smooth muscle cells, trachea mesenchymal cells,	Yamanoi et al, 2019[41]; Ota et al, 2021[42]

	liver Kupffer cells, lung myeloid cells, bladder cells, heart fibroblasts, limb muscle mesenchymal stem cells, pancreatic PP cells, mammary gland luminary epithelial cells, mammary gland stromal cells, pancreatic delta cells, bone marrow immature B cells, pancreatic beta cells, mammary gland basal cells, skin leukocytes, skin keratinocyte stem cells, pancreatic alpha cells, pancreas leukocytes, brain neurons, large intestine enterocytes, pancreatic endothelial cells, brain Bermann glial cells, pancreatic stellate cells	
Frmpd4<br>(a multi-domain protein that regulates dendritic spine morphogenesis/density and is required for the maintenance of excitatory synaptic transmission)	None	Lee et al, 2008[43]; Piard et al, 2018[44]
Prps2<br>(catalyzes the synthesis of phosphoribosyl-pyrophosphate that is essential for nucleotide synthesis)	Bone marrow Slamf1-positive multipotent progenitor cells, bone marrow late pro-B cells, lung epithelial cells, bone marrow common lyphoid progenitor cells, bone marrow pre-natural killer cells, thymus immature T cells, bone marrow megakaryocyte-erythroid progenitor cells, bone marrow granulocyte monocyte progenitor cells, bone barrow macrophages, bone marrow immature B cells, bone marrow Slamf1-negative multipotent progenitor cells, skin epidermal stem cells, bone marrow immature T cells, tongue basal epidermal cells, bone marrow mature natural killer cells, bone marrow hematopoetic precursor cells, kidney collecting duct epithelial cells, thymus DN1 pro-T cells, pancreatic ductal cells, skin	Lei et al, 2015[45]; Lei et al, 2020[46]; Yang et al, 2022[47]

	leukocytes, heart professional antigen-presenting cells, bone marrow B cells, brain oligodendrocytes, large intestine epithelial cells, liver natural killer cells, large intestine goblet cells, trachea blood cells, tongue keratinocytes, bone marrow basophils, kidney macrophages, pancreas endothelial cells, bone marrow immature NK T cells, skin epidermal cells, bladder urothelial cells, pancreatic delta cells, pancreatic leukocytes, liver hepatocytes	
Tlr7 (endosomal receptor that plays a key role in innate and adaptive immunity)	Bone marrow macrophages, lung monocytes, brain microglia, lung classical monocytes, brain macrophages, heart leukocytes, kidney macrophages, pancreas leukocytes, limb muscle macrophages, trachea blood cells, fat myeloid cells	Pisitkun et al, 2006[48]; Subramanian et al, 2006[49]; Fairhurst et al, 2008[50]; Brown et al, 2022[51]
Tlr8 (endosomal receptor that plays a key role in innate and adaptive immunity)	Heart leukocytes, bone marrow monocytes, lung classical monocytes, brain macrophages, skin leukocytes	Guiducci et al, 2013[52]; Kimura et al, 2014[53]; Davidson et al, 2021[54]
Tmsb4x (Plays an important role in the organization of the cytoskeleton by binding to and sequestering actin monomers.)	Lung leukocytes, lung myeloid cells, bone marrow granulocytes, lung natural killer cells, lung monocytes, bone marrow immature natural killer cells, bone marrow basophils, heart professional antigen-presenting cells, limb muscle lymphocytes, spleen macrophages, limb muscle macrophages, thymus DN1 pro-T cells, lung classical monocytes, fat natural killer cells, bone marrow monocytes, fat myeloid cells, bone marrow mature natural killer cells, heart leukocytes, bone marrow granulocytopoetic cells, bone marrow immature NK T cells, bone marrow B cells, liver Kupffer cells, lung T cells,	Wirsching et al, 2014[55]

	liver natural killer cells, bone marrow regulatory T cells, limb muscle B cells, spleen T cells, brain macrophages, pancreatic leukocytes, lung B cells, kidney macrophages, bone marrow immature T cells, trachea blood cells, limb muscle T cells, bone marrow pre-natural killer cells, fat T cells, fat B cells, bone marrow macrophages, heart endothelial cells, kidney leukocytes, spleen B cells, limb muscle endothelial cells, bone marrow naive B cells, thymus leukocytes, trachea endothelial cells, bone marrow immature B cells, bone marrow granulocyte monocyte progenitor cells, fat endothelial cells, brain microglia, brain endothelial cells, mammary gland endothelial cells, lung epithelial cells, liver B cells, bladder urothelial cells, skin leukocytes, fat cells, heart endothelial cells, bone marrow precursor B cells, thymus immature T cells, pancreas endothelial cells, lung endothelial cells, skin keratinocyte stem cells, mammary gland stromal cells, bone marrow late pro-B cells, mammary gland luminal epithelial cells, skin basal epidermal cells, liver hepatic sinusoidal endothelial cells, fat mesenchymal stem cells, skin epidermal stem cells, heart myofibroblast cells, limb muscle mesenchymal stem cells, heart cells, lung cells, bone marrow megakaryocyte erythroid progenitor cells, bone marrow Slamf1-negative multipotent progenitor cells, skin epidermal cells, brain pericytes, limb muscle satellite stem cells, kidney endothelial cells, heart fibroblasts, brain oligodendrocytes, tongue basal	

	epidermal cells, lung stromal cells, trachea mesenchymal cells, mammary gland basal cells, bladder cells, bone marrow Slamf1-positive multipotent progenitor cells, pancreatic stellate cells, bone marrow hematopoietic precursor cells, pancreatic alpha cells, bone marrow common lymphoid progenitor cells, brain Bergmann glial cells, brain oligodendrocyte precursor cells, pancreatic ductal cells, brain neurons, large intestine epithelial cells, trachea epithelial cells, large intestine goblet cells, pancreatic PP cells, tongue keratinocytes, lung ciliated columnar cells of tracheobronchial tree, heart smooth muscle cells, pancreatic delta cells, pancreatic type B cells, heart erythrocytes, heart cardiac muscle cells, brain astrocytes, large intestine enteroendocrine cells, large intestine epithelial brush cells, kidney collecting duct cells, pancreatic acinar cells, liver hepatocytes, kidney proximal tubule epithelial cells	

Supplementary Figures

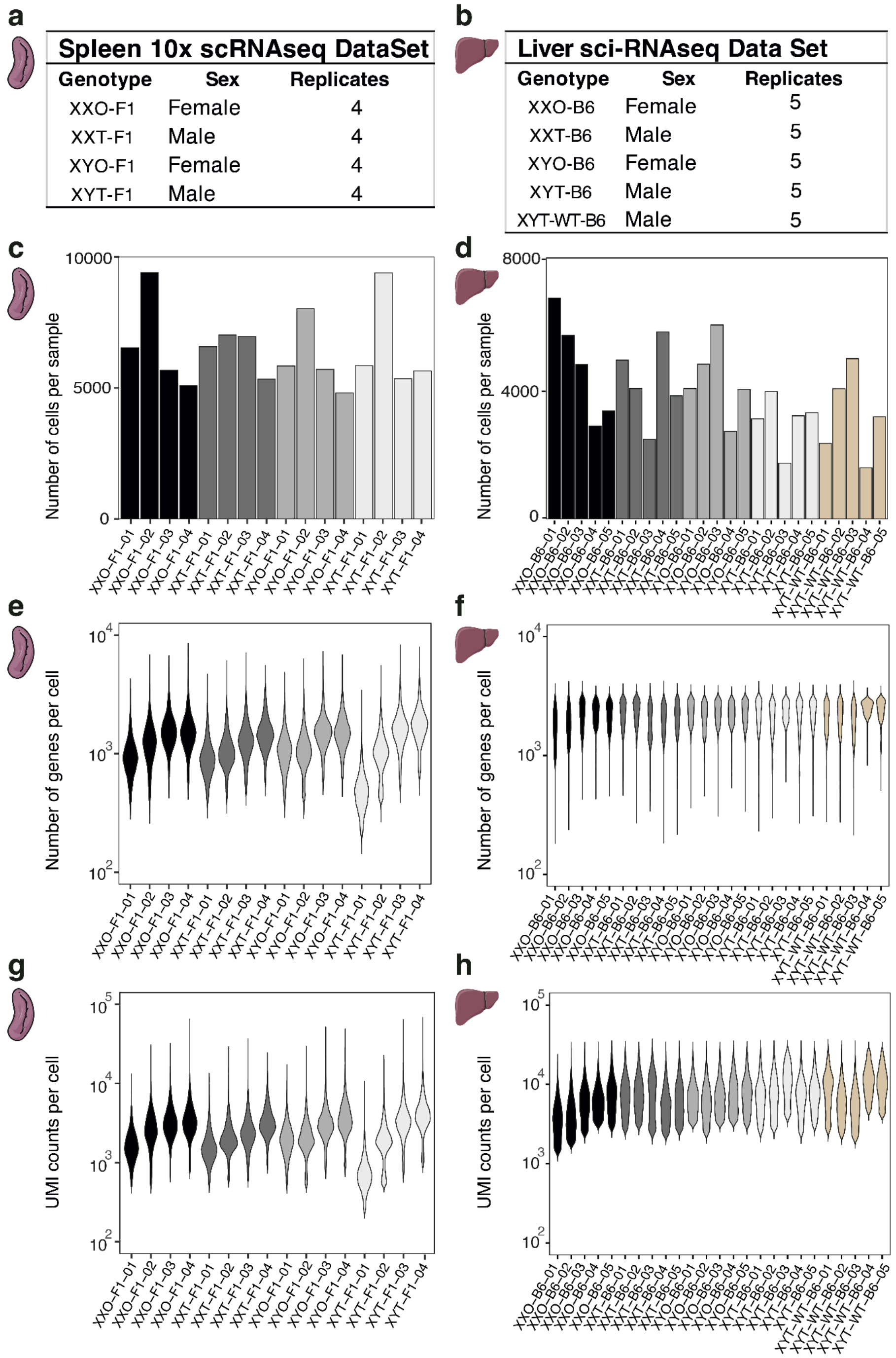

Supplementary Figure 1: Quality control of the single cell and single nucleus RNA-sequencing datasets. a-b, Overview of samples used**. c-d,** Number of cells/nuclei recovered per biological replicate. **e-f,** Median number of genes per cell/nucleus in each biological replicate. **g-h,** Median number of UMIs (Unique Molecular Identifiers) per cell/nucleus in each biological replicate. The number of cells/nuclei, genes and UMIs are consistent between the different replicates. The spleen and liver icons were created using BioRender.com.

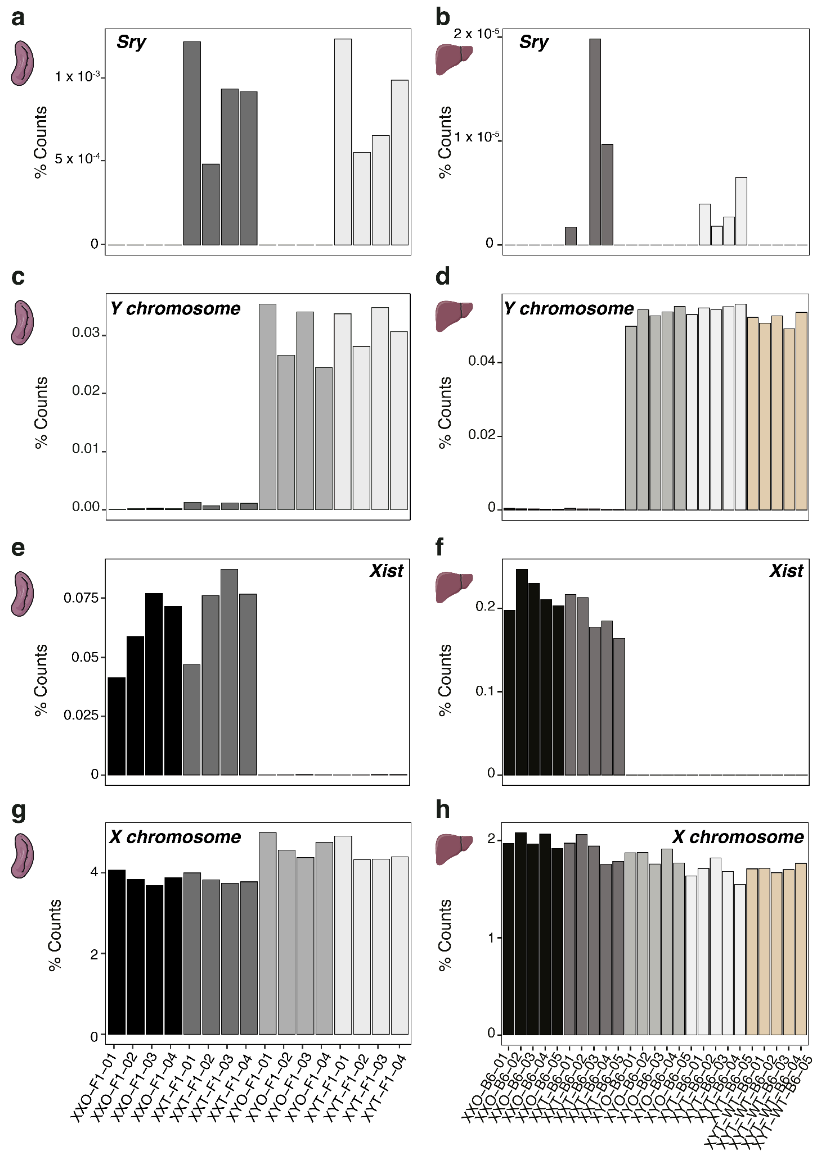

Supplementary Figure 2: Genotyping of the mice by transcriptomic data. a-b, *Sry* expression in each biological replicate calculated as a percentage of *Sry* counts / Total counts**.** The data confirmed the presence of *Sry* in the FCG gonadal male mice (XYT and XXT). **c-d** Y chromosome expression in each biological replicate calculated as a percentage of Y chr. counts / Total counts. Y chromosome counts are only present in the mice carrying a Y chromosome. **e-f,** *Xist* expression in each biological replicate calculated as a percentage of Xist counts / Total counts. As expected, *Xist* is expressed only in mice carrying two X chromosomes. **g-h,** X chromosome expression in each biological replicate calculated as a percentage of X chr. counts / Total counts. In spleen (**g**) the counts are higher in XY genotypes, because of the high expression of *Tmsb4x*. In liver (**h**) the counts are comparable between XX and XY, presumably because of proportionally fewer immune cells highly expressing the translocated genes. The spleen and liver icons were created using BioRender.com.

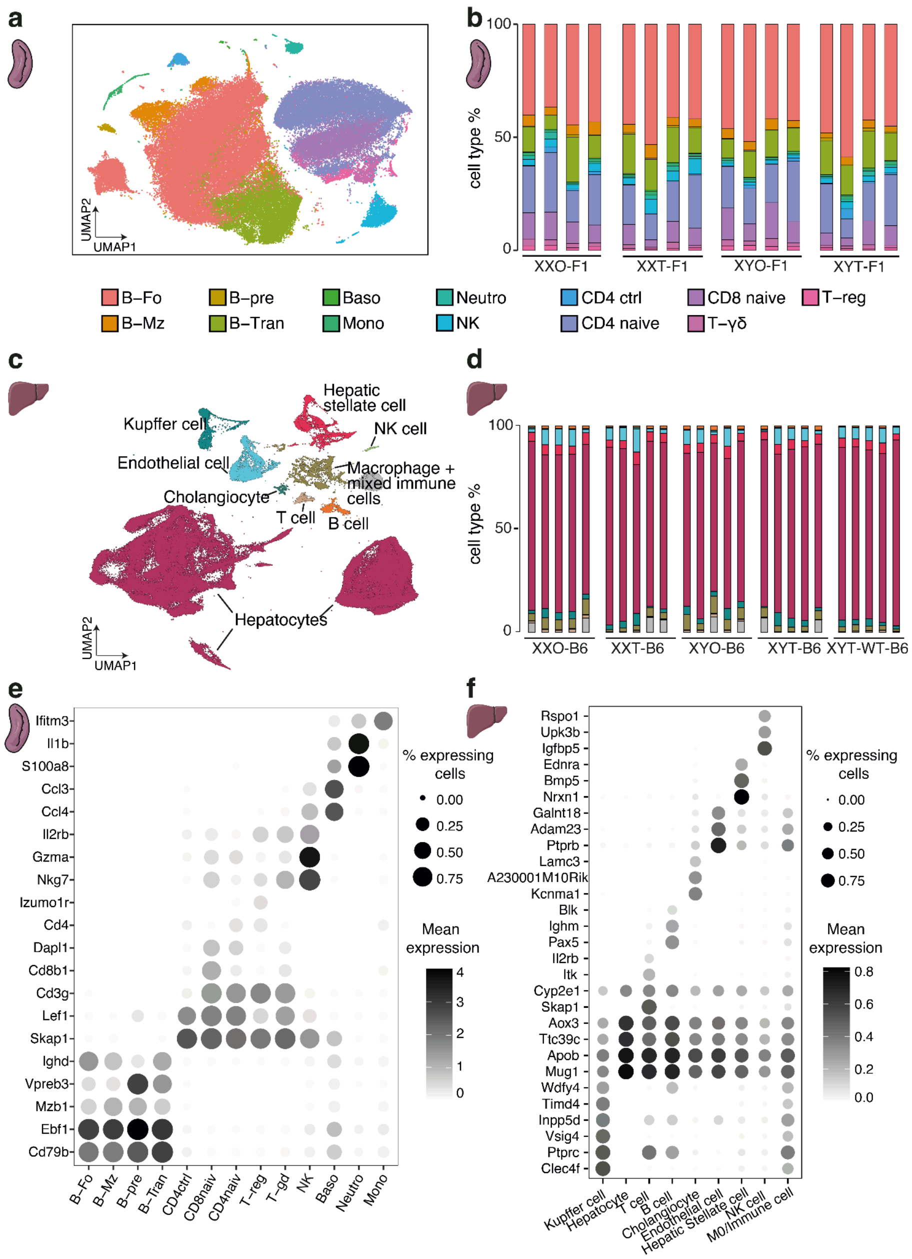

Supplementary Figure 3: Cell types annotation. a-c, Uniform manifold approximation and projection (UMAP) plots of the sequenced spleen cells (a) and liver nuclei (c). Cells are coloured based on cell type. B-Fo: B-Follicular; B-Mz: B-Marginal Zone; B-Tran: B-Transitional; Baso: Basophil; Mono: Monocyte; Neutro: Neutrophil; NK: Natural Killer; CD4 ctrl: CD4 Control; T-γδ: T-Gamma-Delta; T-reg: T-regulatory. **b-d,** Barplots showing % of each cell type for each biological replicate. **e-f,** Cluster and cell type-specific genes used to annotate cell types. For the spleen dataset, 103,000 cells from 16 individual mice were analysed with 13 unique cell types annotated. Consistent with previous reports, the splenic immune cell population of mice is dominated by lymphoid lineage cells. For the liver dataset, 147,888 cells from 25 individual mice were analysed, with 9 unique cell types identified. The spleen and liver icons were created using BioRender.com.

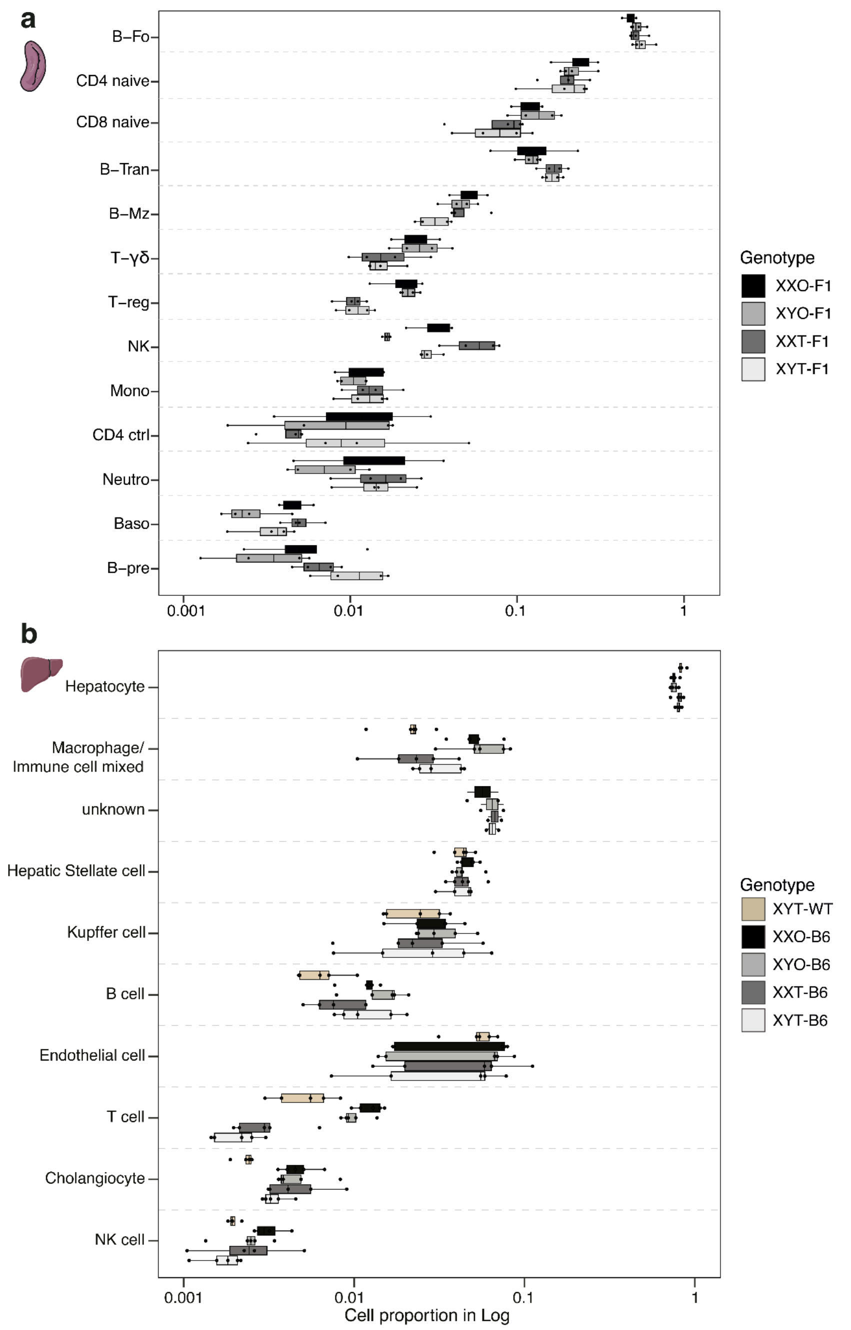

Supplementary Figure 4: Few changes in cell type proportions across FCG genotypes. Cell type proportions comparison between the different genotypes in the spleen (**a**) and liver (**b**) dataset. Changes in cell type proportions between the FCG genotypes are small and primarily driven by differences in gonad type. Median and standard deviation are represented. B-Fo: B-Follicular; B-Mz: B-Marginal Zone; B-Tran: B-Transitional; Baso: Basophil; Mono: Monocyte; Neutro: Neutrophil; NK: Natural Killer; CD4 ctrl: CD4 Control; T-γδ: T-Gamma-Delta; T-reg: T-regulatory. The spleen and liver icons were created using BioRender.com.

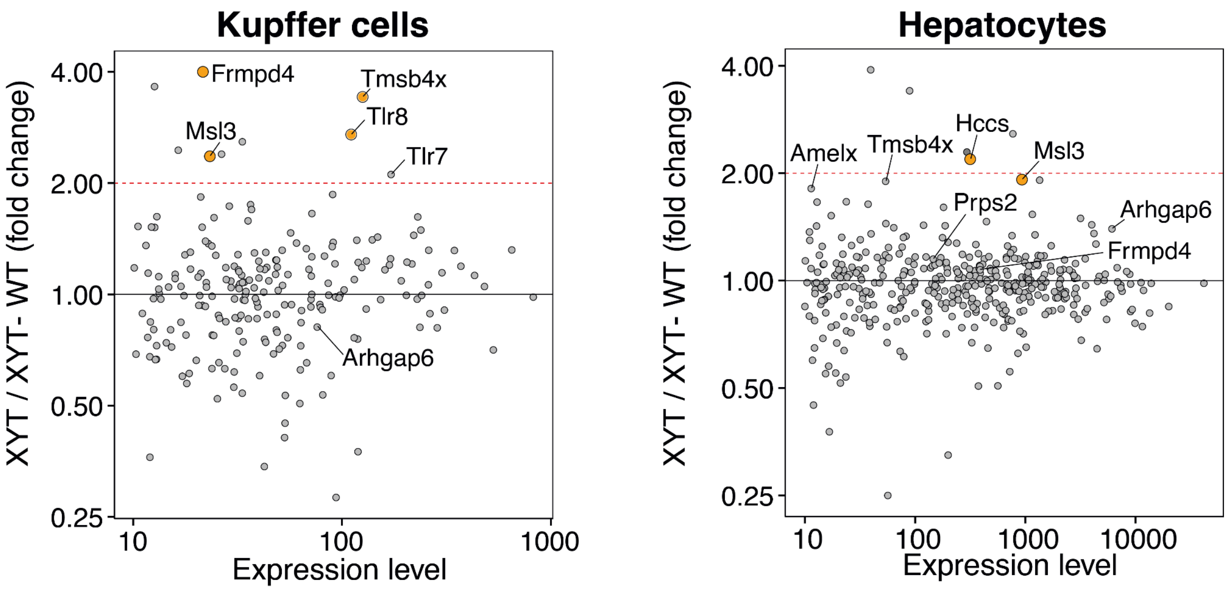

Supplementary Figure 5: X chromosome genes comparison between FCG XYT and XYT-WT liver nuclei. Differential expression analysis of X chromosome genes on pseudo-bulked single-nucleus RNA sequencing of liver Kupffer cells and hepatocytes isolated from B6 FCG XYT and XYT-WT mice. The x-axis indicates the expression level as total read counts. Highlighted in orange are all genes in the PAR-adjacent region that have a positive fold change and an adjusted p-value < 0.1 (DESeq2 Wald test).

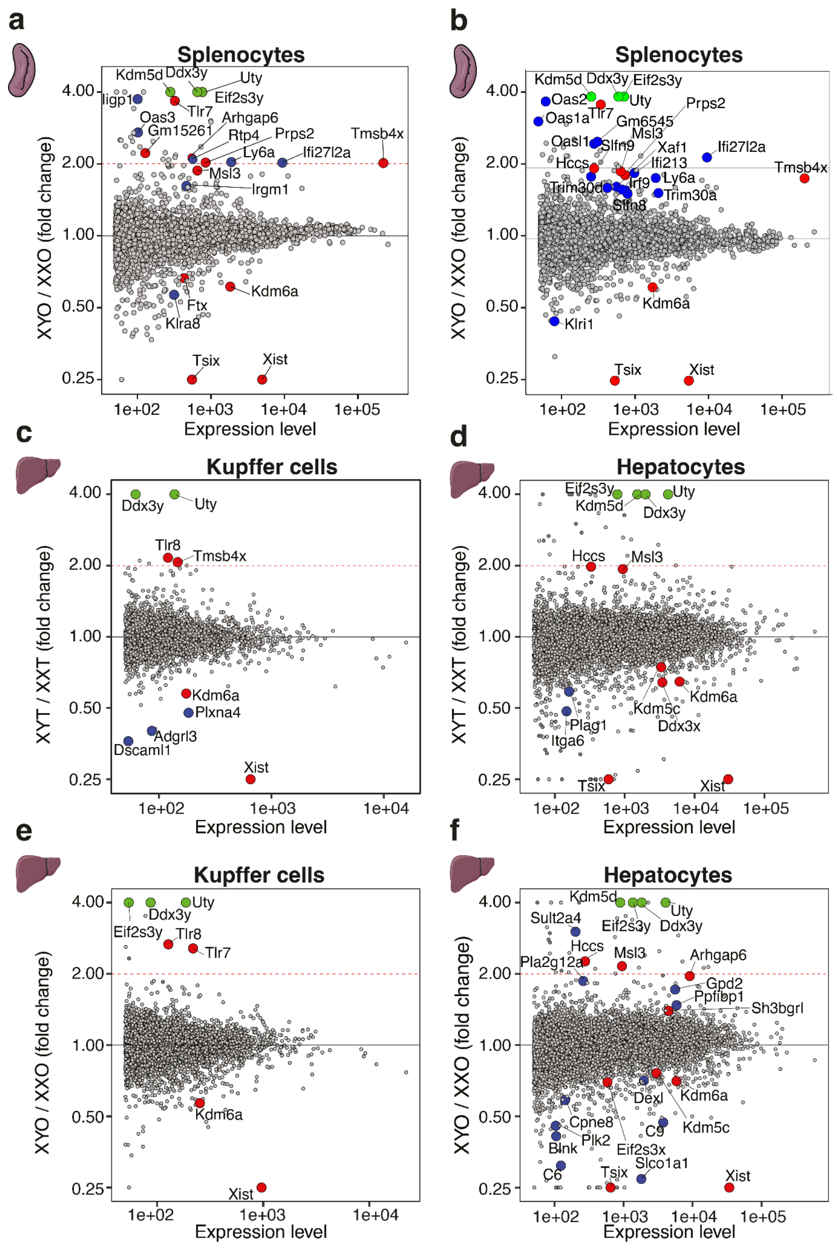

Supplementary Figure 6: The increased expression of the translocated genes has a minor effect on autosomal gene expression. a-b, Differential expression analysis on pseudo-bulked single-cell RNA sequencing of splenocytes between (**a**) XYO and XXO mice and between (**b**) XYT and XXT mice. **c-f,** Differential expression analysis on pseudo-bulked single-nucleus RNA sequencing of liver (**c-e**) Kupffer cells and (**d-f**) hepatocytes between XYO and XXO mice and between XYT and XXT mice The x-axis indicates the expression level as total read counts. The y-axis are capped at 4 and 0.25, genes such as *Xist, Uty, Kdm5d* have greater FC than 4 or 0.25. The coloured dots represent genes with 2 fold change and an adjusted p-value < 0.1 (DESeq2 Wald test). Red dot: X chromosome genes. Green dot: Y chromosome genes. Blue dot: autosomal genes. Some autosomal genes, in particular *Ly6a* and *Ifi27l2a,* which we detect as differentially expressed in splenocytes in both comparisons are interferon target genes, potentially downstream of *Tlr7*. The spleen and liver icons were created using BioRender.com.

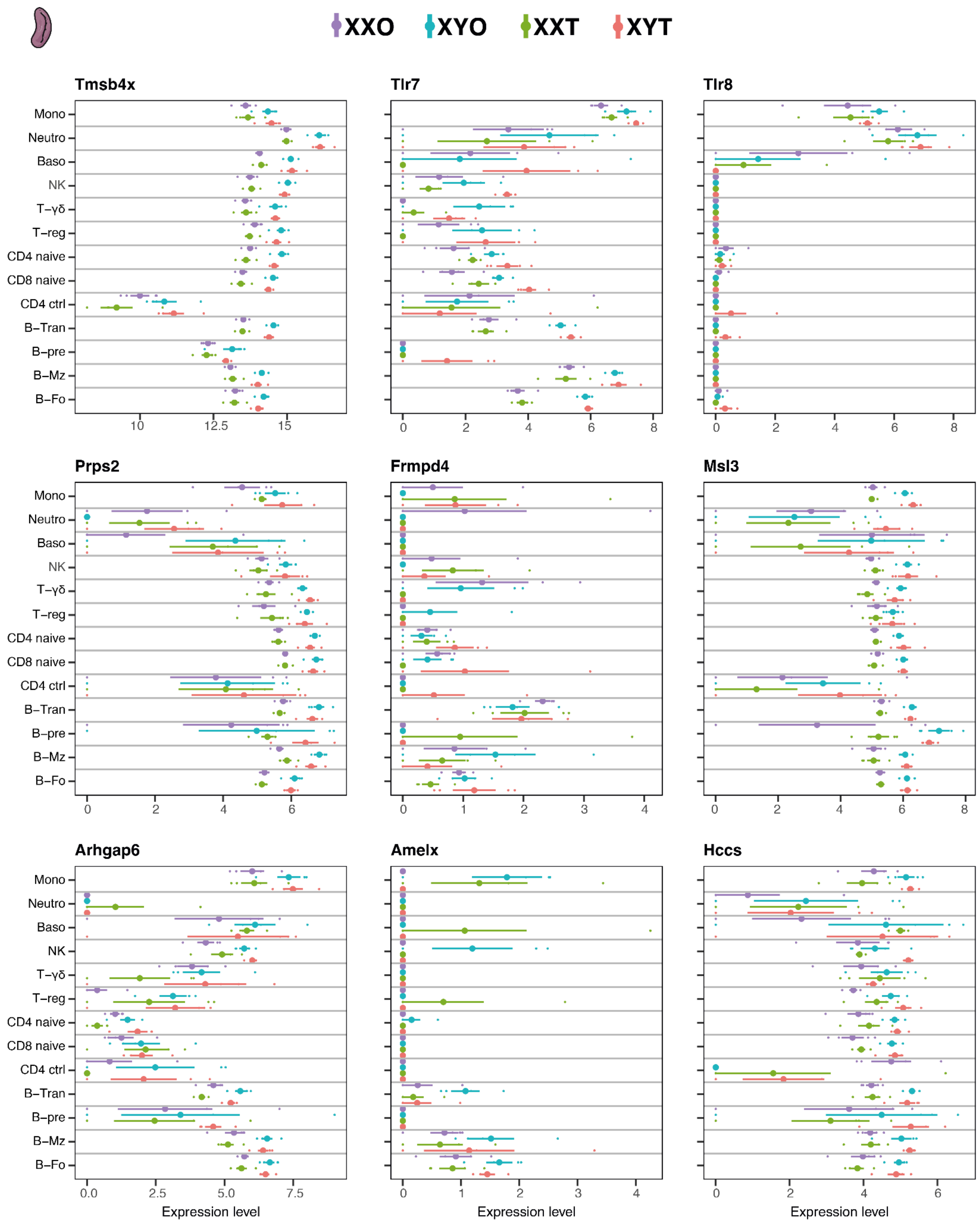

Supplementary Figure 7: Cell type-specific expression of the translocated genes in splenocytes. Independent measurements of all the biological replicates are shown as small dots. The main dot represents the mean of normalised expression with the standard error. The translocated genes show cell-type specific expression and mostly a higher expression in XY genotypes. B-Fo: B-Follicular; B-Mz: B-Marginal Zone; B-Tran: B-Transitional; Baso: Basophil; Mono: Monocyte; Neutro: Neutrophil; NK: Natural Killer; CD4 ctrl: CD4 Control; T-γδ: T-Gamma-Delta; T-reg: T-regulatory. The spleen icons were created using BioRender.com.

a

Sample Name	Genotype	Sex	Tissue type	Genetic background
XYT-WT-B6	XY	Male	Liver	C57BL/6J
XYT-B6-founder 1	XY	Male	Earpunch	Four Core - C57BL/6J
XYT-B6-founder 2	XY	Male	Earpunch	Four Core - C57BL/6J
XYT-WT-F1	XY	Male	Liver	C57BL/6J x CAST/EiJ
XYT-F1	XY	Male	Liver	Four Core - C57BL/6J x CAST/EiJ
XXT-F1	XX	Male	Liver	Four Core - C57BL/6J x CAST/EiJ
XYO-F1	XY	Female	Liver	Four Core - C57BL/6J x CAST/EiJ
XXO-F1	XX	Female	Liver	Four Core - C57BL/6J x CAST/EiJ

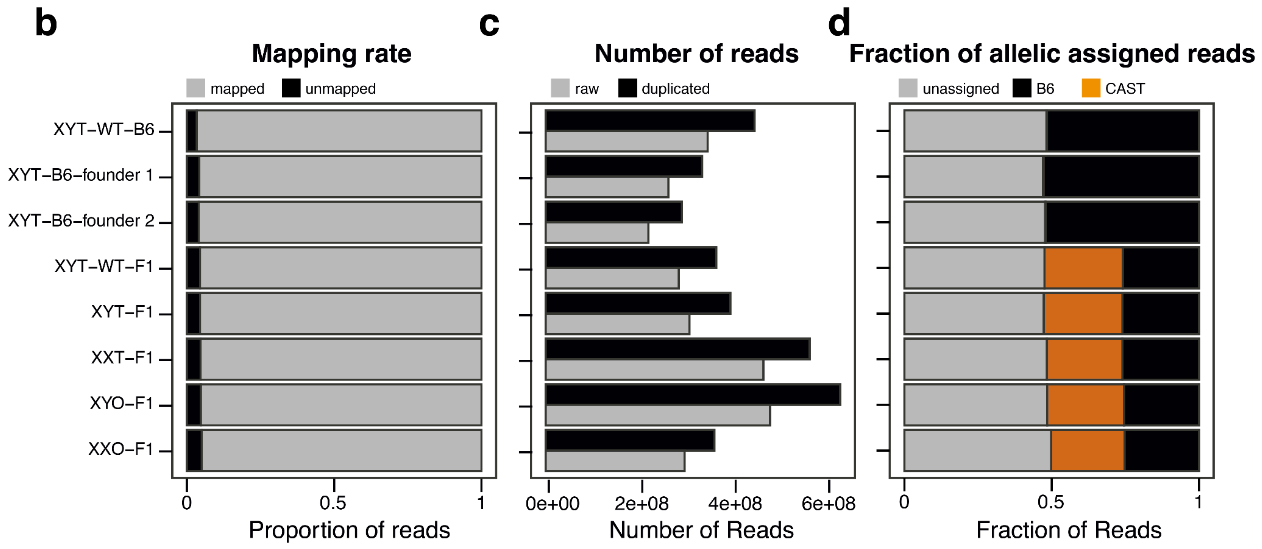

Supplementary Figure 8: Quality control of the whole genome sequencing data. a, Summary table of the samples used for whole-genome sequencing analysis in Supplementary Figures 9-11. **b,** Barplots showing the mapping rate across samples. **c,** Barplots showing the number of mapped reads, before and after duplicate exclusion. **d,** Barplots showing the fraction of reads assignable to the C57BL/6J and CAST/EiJ haplotypes based on known heterozygous single-nucleotide variants.

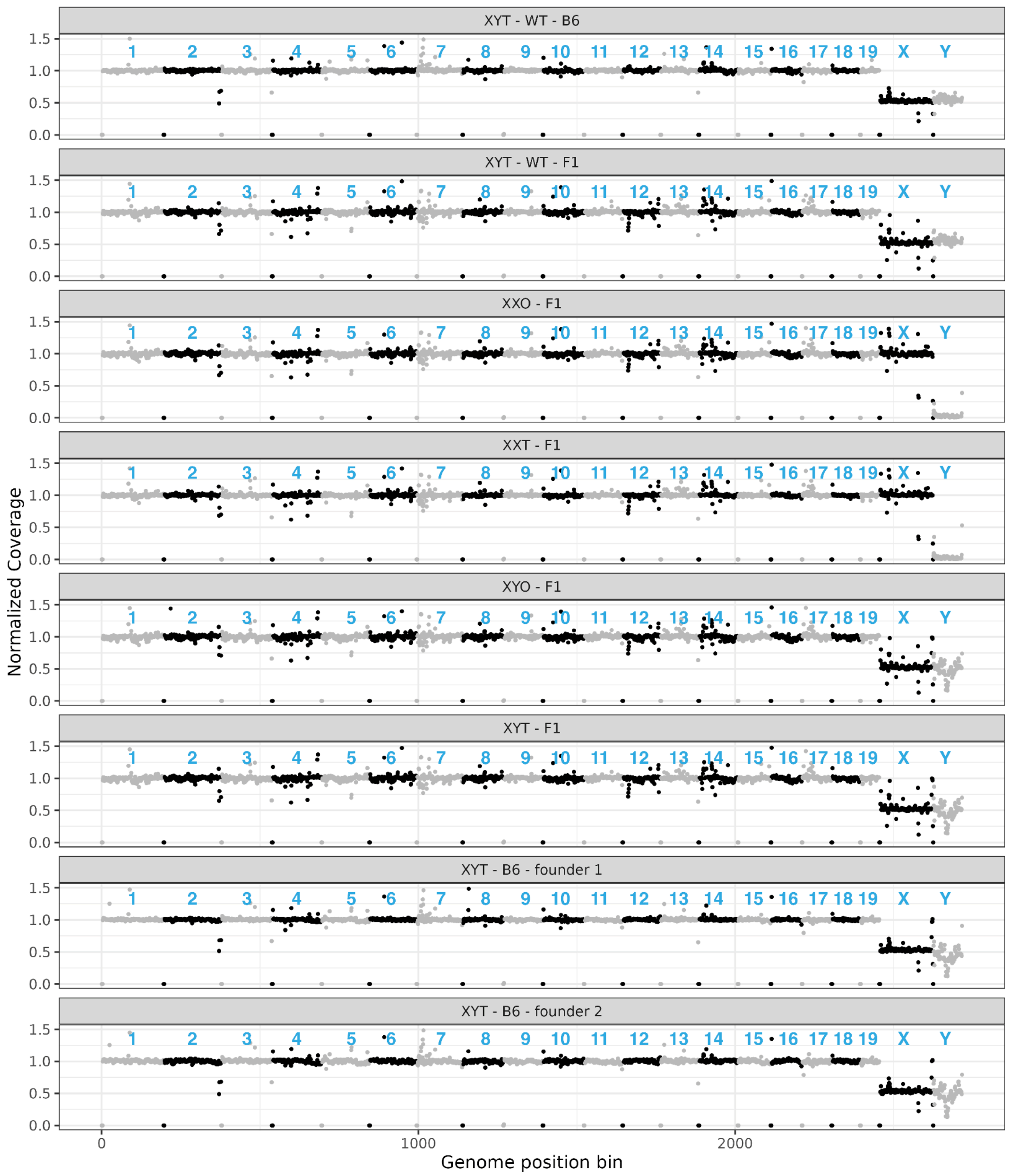

Supplementary Figure 9: Genome-wide coverage plots across sequenced samples. Coverage is computed as the number of deduplicated, mapped reads in 1 mega-base windows, normalized to the median across the sample corresponding to a diploid genome. Blue labels denote the individual chromosome represented by the alternating black and grey segments.

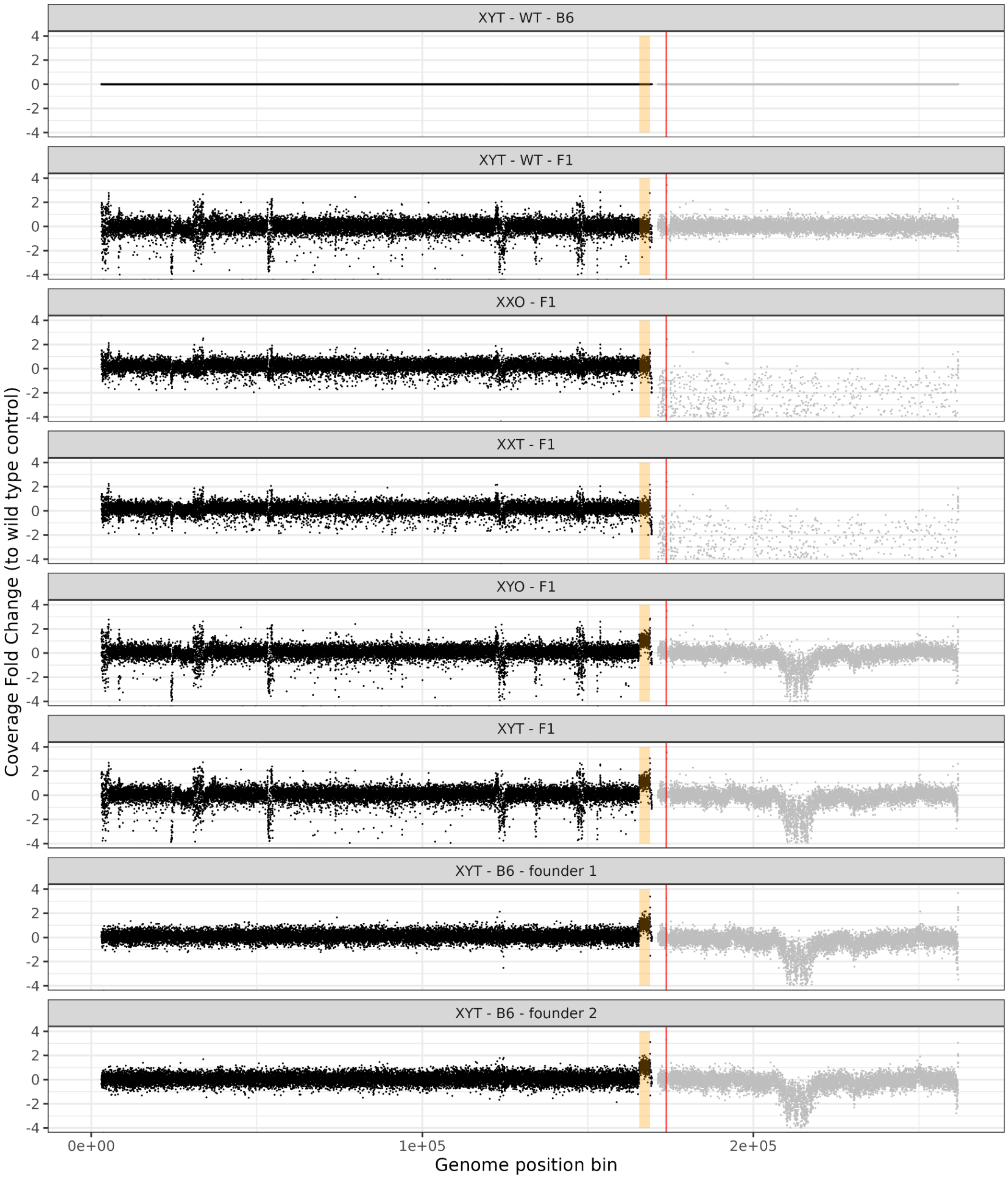

Supplementary Figure 10: Sex-chromosome coverage plots across sequenced samples. Coverage is computed as the number of deduplicated, mapped reads in 1 mega-base window, and normalized to the median across the sample, corresponding to a diploid genome. The X-chromosome is shown in black, the Y-chromosome in grey. In orange, the putative duplicated region is highlighted. In red, the location of the *Sry* gene is shown.

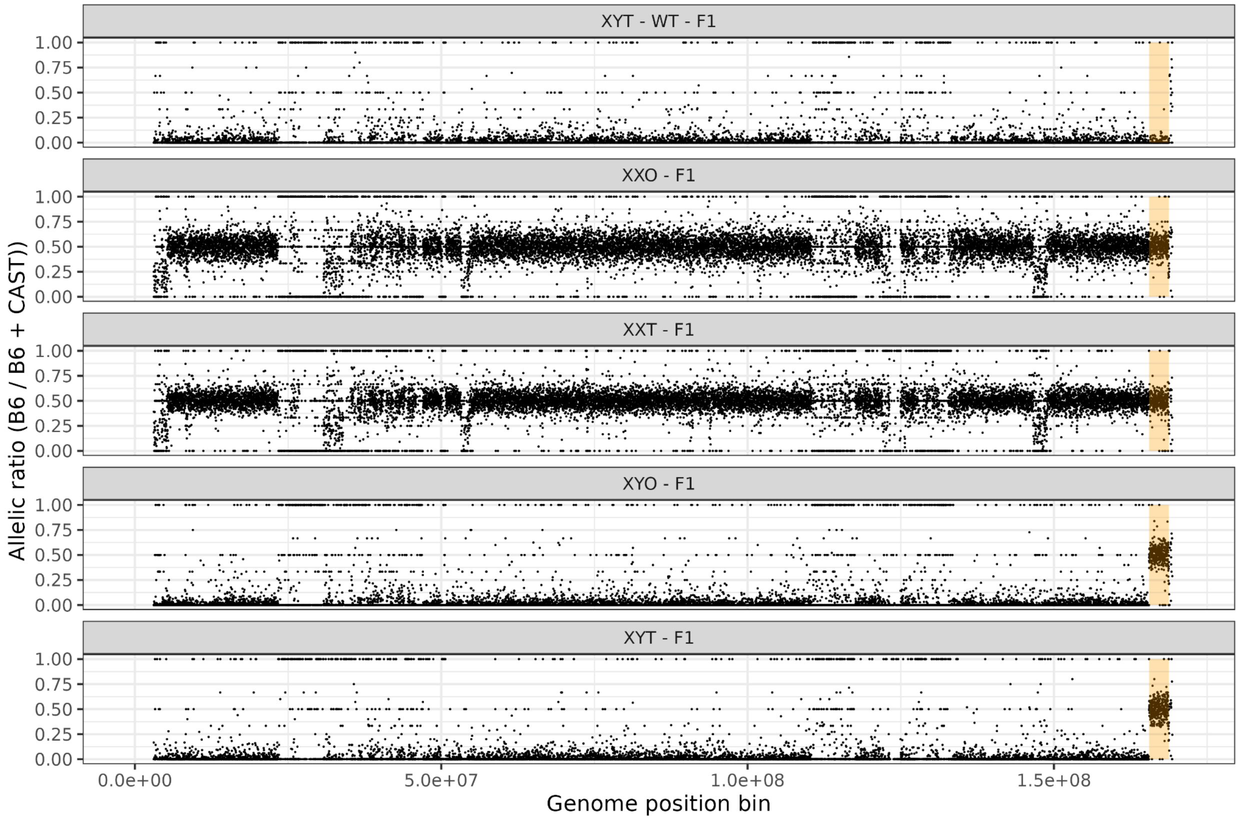

Supplementary Figure 11: Allele-specific coverage plots on the X-chromosome across sequenced samples. Reads are assigned to paternal (B6) or maternal (CAST) haplotypes based on known homozygous variants between the two strains. Coverage per haplotype is computed as the number of deduplicated, mapped reads in 1 mega-base windows and allelic ratio B6 / (B6 + CAST). In orange, the putative duplicated region is highlighted. As the X-chromosome is maternally inherited, the presence of B6 X-chromosomal reads in XY mice demonstrates a transfer of X-chromosomal material to a different chromosome.

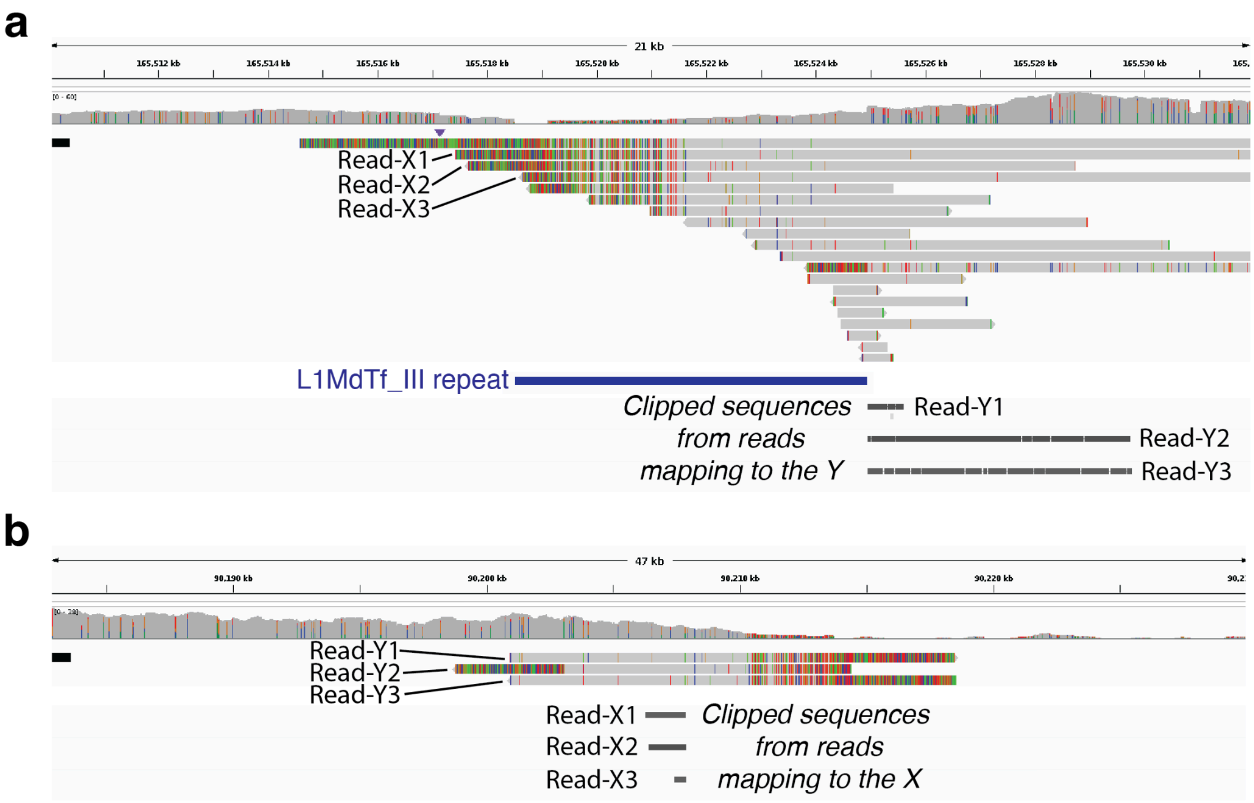

Supplementary Figure 12: Fine-mapping of the translocation breakpoint using long-read sequencing data. a, IGV browser screenshot showing long-read Nanopore sequencing from a F1 B6xCAST-FCG-XYT hybrid individual at the putative translocation break point on the X-chromosome, which overlaps a LINE repeat sequence (L1MdTf_III family). The coverage track shows homozygous SNPs before (indicated in red and blue) and heterozygous SNPs (each line is half red and half green) after the breakpoint. B6 reads (paternal haplotype, from the translocated sequence) are shown and map to the repeat and in some cases carry soft-clipped (i.e. unaligned on the X-chromosome) sequences that map to the same position on the Y-chromosome (see **b**, below). Below, clipped sequences from three reads mapping to the Y-chromosome are shown. **b,** As **a**, showing the Y-chromosomal locus that clipped sequences from reads in **a** map to. Three reads mapping to the Y-chromosome are shown that show partial mapping to the putative translocation locus on the X-chromosome. These reads support the presence of the translocation, and the overlap with a repeat sequence suggests that the translocation might have originated through recombination with a homologous repeat on the Y.

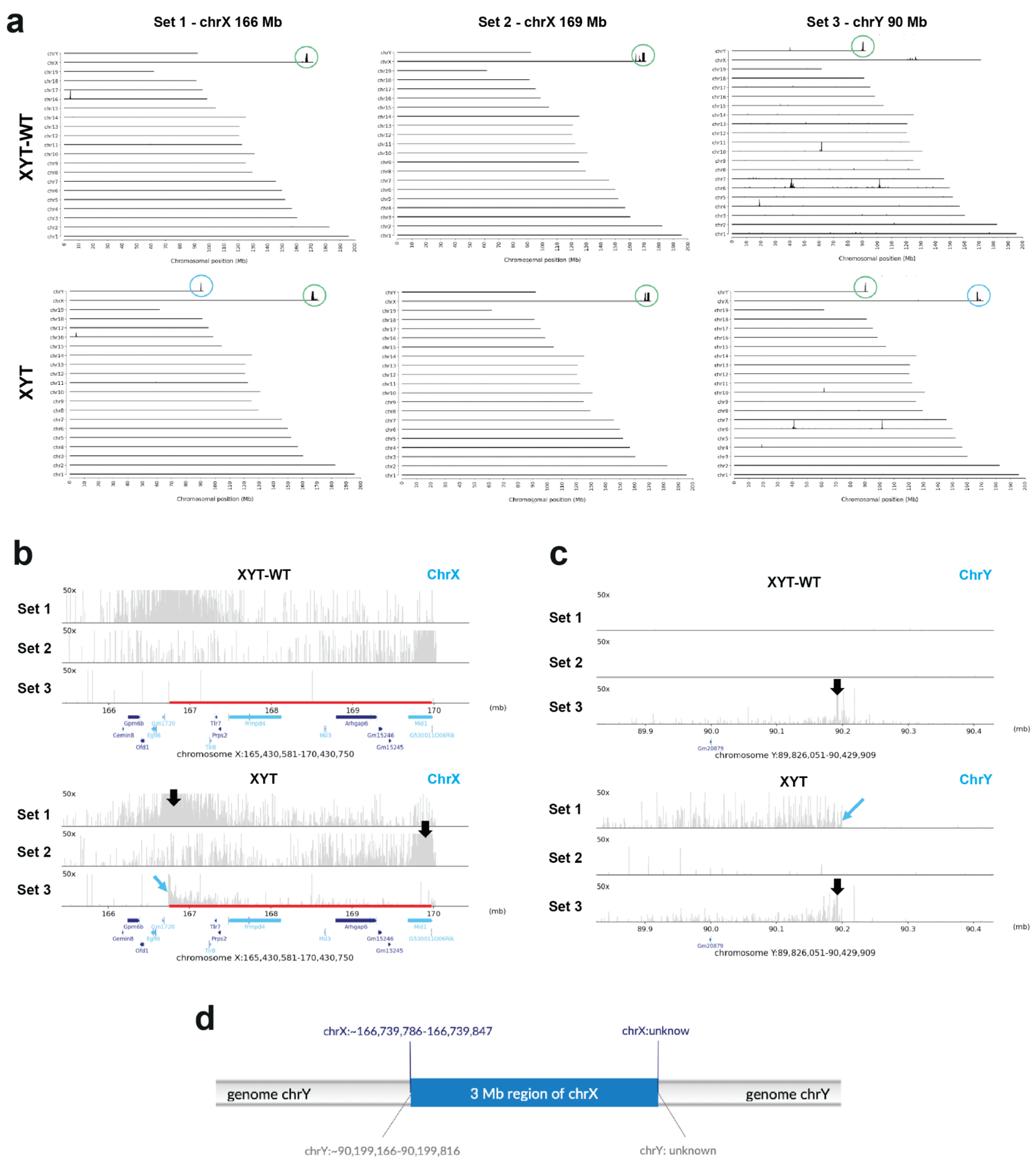

Supplementary Figure 13: Targeted Locus Amplification (TLA) analysis of the origin and destination of the translocation. a, TLA sequencing coverage across the whole genome for three primer pairs targeting the 5' end of the chrX duplication (set 1), 3' end of the chrX duplication (set 2) and the chrY region identified as probable translocation destination using set 1 (set 3) in XYT and XYT-WT splenocytes. Green circles represent primer locations and blue circles represent the translocation destination (set 1) and origin (set 3). Autosomal peaks are likely caused by the high homology of the primer target region to many other regions of the genome. **b,** TLA sequencing coverage across chrX:165,430,581-170,430,750. Black arrows represent primer locations, the blue arrow represents the location of the chrX-chrY breakpoint and the red bar indicates the expected 3 Mb translocated region. The Y-axis is limited to 50x. **c,** TLA sequencing coverage across chrY:89,826,051-90,429,909. Black arrows represent the primer location and the blue arrow represents the location of the chrX-chrY breakpoint. The Y-axis is limited to 50x. **d,** Schematic representation of the translocation destination based on TLA data.

Sample Name	Genotype	Sex	Genetic background	Translocation	Samples collected
XYT-WT-B6-Ld	XY	Male	C57BL/6J	No	2023
XYT-B6-Ld	XY	Male	Four Core - C57BL/6J	No	2023
XYT-MF1-Ld	XY	Male	Four Core - MF1	No	2023
XYO-B6-Ld	XY	Female	Four Core - C57BL/6J	No	2023
XYO-MF1-Ld	XY	Female	Four Core - MF1	No	2023
XYT-B6-Cal	XY	Male	Four Core - C57BL/6J	Yes	2023
XYO-B6-Cal	XY	Female	Four Core - C57BL/6J	Yes	2023
XYT-B6-Cal2	XY	Male	Four Core - C57BL/6J	Yes	2010

Supplementary Table 3: Summary table of the samples used for whole-genome sequencing analysis of Supplementary Figures 14 and 15.

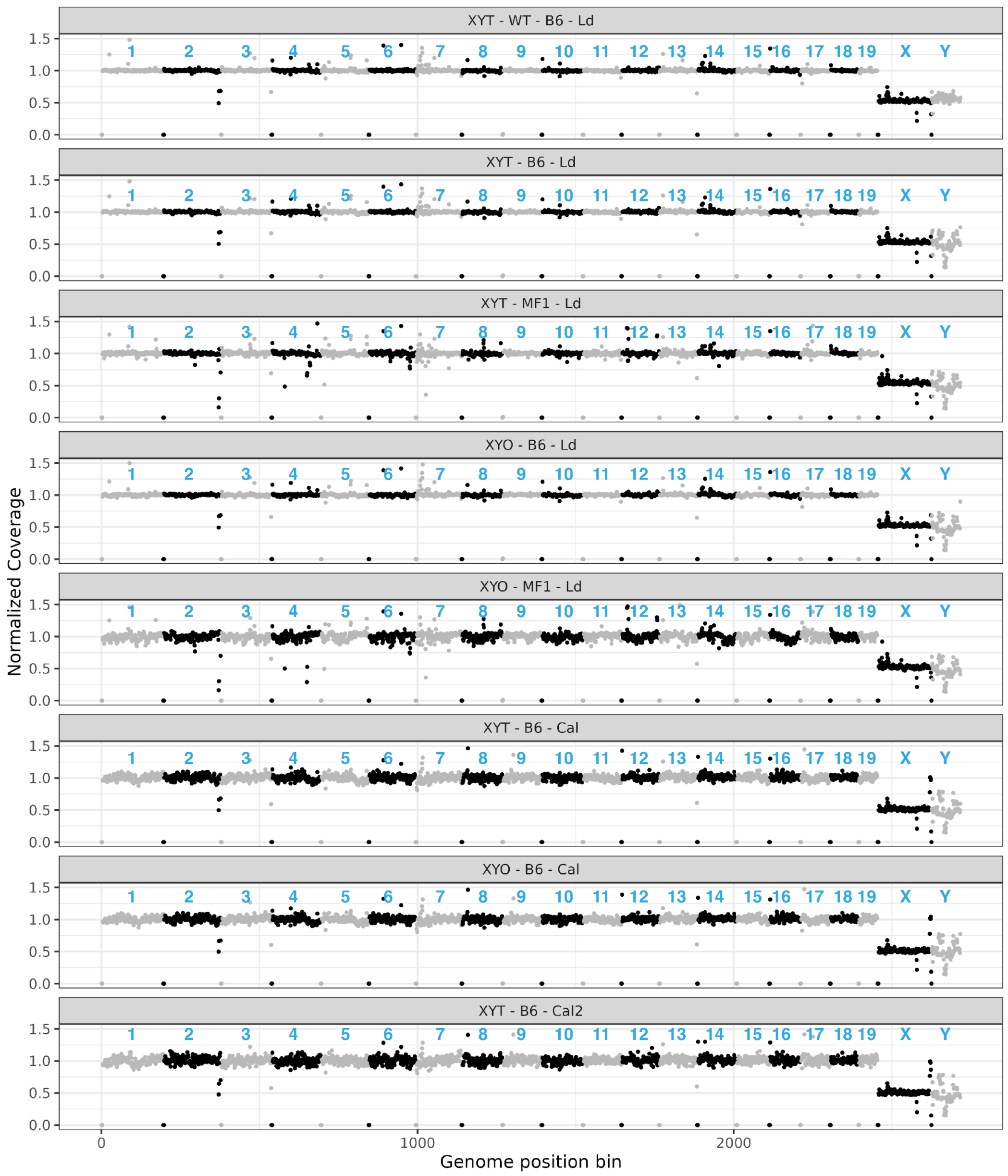

Supplementary Figure 14: Genome-wide coverage plots across sequenced samples from separate colonies (Crick Institute in London, Ld; and UCLA in California, Cal). Coverage is computed as the number of deduplicated, mapped reads in 1 mega-base windows, and normalized to the median across the sample, corresponding to a diploid genome. Blue labels denote the individual chromosome represented by the alternating black and grey segments, the last two chromosomes are X (black) and Y (grey).

These data support the conclusion that the translocation in the B6-FCG-XYT-Cal mice, which were donated to Jackson Laboratory in 2010 as strain 10905, has been present since at least 2010. The independent FCG colonies at the Crick Institute, both MF1 and B6, do not have this translocation. The Crick Institute's MF1 FCG mice were sent to the Arnold laboratory at UCLA in 2000, and the translocation appears to have occurred between 2000 and 2010 during backcrossing of the FCG model to B6.

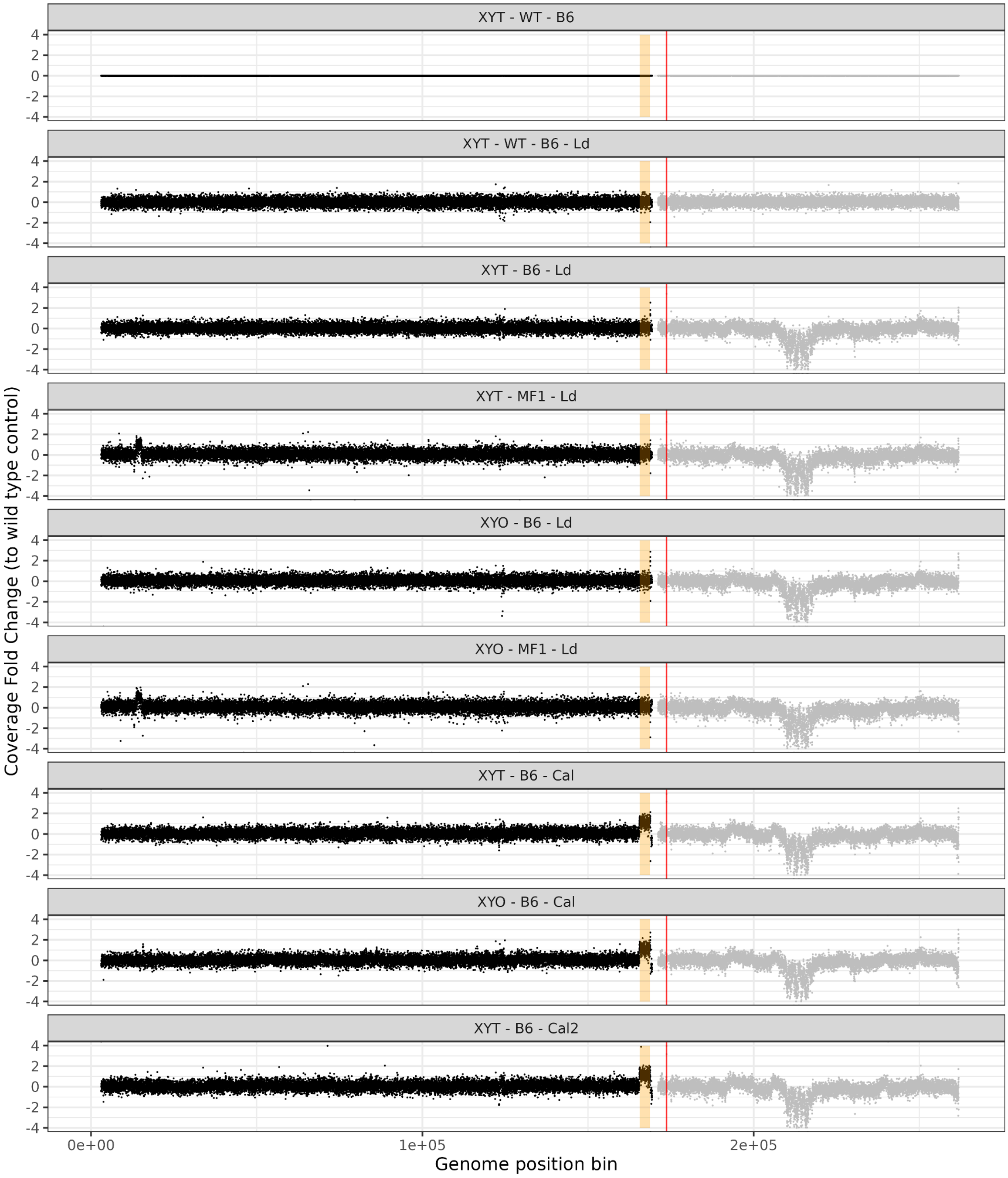

Supplementary Figure 15: Sex-chromosome coverage plots across sequenced samples from separate colonies (Crick in London, Ld; and UCLA in California, Cal). Coverage is computed as the number of deduplicated, mapped reads in 1 mega-base windows, and normalised to the median across the sample, corresponding to a diploid genome. The X-chromosome is shown in black, the Y-chromosome in grey. In orange, the putative duplicated region is highlighted. In red, the location of the *Sry* gene is shown. The Ld colonies do not show the translocation, whereas the Cal mice do.

35. Cho, E. S. *et al.* Alteration of conserved alternative splicing in AMELX causes enamel defects. *J. Dent. Res.* **93**, 980–987 (2014).

36. Miao, X. *et al.* Stage-Specific Role of Amelx Activation in Stepwise Ameloblast Induction from Mouse Induced Pluripotent Stem Cells. *Int. J. Mol. Sci.* **22**, (2021).

37. Prakash, S. K. *et al.* Functional analysis of ARHGAP6, a novel GTPase-activating protein for RhoA. *Hum. Mol. Genet.* **9**, 477–488 (2000).

38. Wu, Y., Xu, M., He, R., Xu, K. & Ma, Y. ARHGAP6 regulates the proliferation, migration and invasion of lung cancer cells. *Oncol. Rep.* **41**, 2281–2888 (2019).

39. Li, P., Lv, H., Xu, M., Zang, B. & Ma, Y. ARHGAP6 Promotes Apoptosis and Inhibits Glycolysis in Lung Adenocarcinoma Through STAT3 Signaling Pathway. *Cancer Manag. Res.* **12**, 9665–9678 (2020).

40. Chen, W. *et al.* ARHGAP6 inhibits bladder cancer cell viability, migration, and invasion via β-catenin signaling and enhances mitomycin C sensitivity. *Hum. Cell* **36**, 786–797 (2023).

41. Yamanoi, K. *et al.* Acquisition of a side population fraction augments malignant phenotype in ovarian cancer. *Sci. Rep.* **9**, 14215 (2019).

42. Ota, R., Hayashi, M., Morita, S., Miura, H. & Kobayashi, S. Absence of X-chromosome dosage compensation in the primordial germ cells of Drosophila embryos. *Sci. Rep.* **11**, 4890 (2021).

43. Lee, H. W. *et al.* Preso, a novel PSD-95-interacting FERM and PDZ domain protein that regulates dendritic spine morphogenesis. *J. Neurosci.* **28**, 14546–14556 (2008).

44. Piard, J. *et al.* FRMPD4 mutations cause X-linked intellectual disability and disrupt dendritic spine morphogenesis. *Hum. Mol. Genet.* **27**, 589–600 (2018).

45. Lei, B. *et al.* PRPS2 Expression Correlates with Sertoli-Cell Only Syndrome and Inhibits the Apoptosis of TM4 Sertoli Cells. *J. Urol.* **194**, 1491–1497 (2015).

46. Lei, B. *et al.* Phosphoribosyl-pyrophosphate synthetase 2 (PRPS2) depletion regulates spermatogenic cell apoptosis and is correlated with hypospermatogenesis. *Asian J. Androl.* **22**, 493–499 (2020).

47. Yang, R. *et al.* Identification of purine biosynthesis as an NADH-sensing pathway to mediate energy

stress. *Nat. Commun.* **13**, 7031 (2022).

48. Pisitkun, P. *et al.* Autoreactive B cell responses to RNA-related antigens due to TLR7 gene duplication. *Science* **312**, 1669–1672 (2006).

49. Subramanian, S. *et al.* A Tlr7 translocation accelerates systemic autoimmunity in murine lupus. *Proc. Natl. Acad. Sci. U. S. A.* **103**, 9970–9975 (2006).

50. Fairhurst, A.-M. *et al.* Yaa autoimmune phenotypes are conferred by overexpression of TLR7. *Eur. J. Immunol.* **38**, 1971–1978 (2008).

51. Brown, G. J. *et al.* TLR7 gain-of-function genetic variation causes human lupus. *Nature* **605**, 349–356 (2022).

52. Guiducci, C. *et al.* RNA recognition by human TLR8 can lead to autoimmune inflammation. *J. Exp. Med.* **210**, 2903–2919 (2013).

53. Kimura, J. *et al.* Overexpression of Toll-like receptor 8 correlates with the progression of podocyte injury in murine autoimmune glomerulonephritis. *Sci. Rep.* **4**, 7290 (2014).

54. Davidson, A. *et al.* Overexpression of Human TLR8 Causes Fatal Anemia in SLE-Prone Mice By Altering the Bone Marrow Erythropoietic Niche. *Blood* **138**, 1989 (2021).

55. Wirsching, H.-G. *et al.* Thymosin β 4 gene silencing decreases stemness and invasiveness in glioblastoma. *Brain* **137**, 433–448 (2014).
